# Artificial Intelligence for Hospital Scheduling: A Simple, Reproducible, and Effective Model

**DOI:** 10.7759/cureus.101412

**Published:** 2026-01-13

**Authors:** Corentin Biot, Tressy Tabbuso, Yoann Marechal

**Affiliations:** 1 Pediatrics, CHU (Centre Hospitalier Universitaire) Charleroi-Chimay, Charleroi, BEL; 2 Neonatology, CHU (Centre Hospitalier Universitaire) Charleroi-Chimay, Charleroi, BEL

**Keywords:** artificial intelligence in medicine, chat gpt, emergency medicine, excel, pediatrics, scheduling

## Abstract

Hospital scheduling, particularly for on-call shifts and daily assignments, is a complex task that must account for numerous factors, such as service requirements, staff preferences, and unplanned absences. Traditional methods often result in significant administrative burden and can lead to staff frustration, potentially affecting the quality of care. This study explores the use of artificial intelligence-based large language models (AI LLMs) to automate hospital scheduling through widely accessible tools, aiming to simplify the process, reduce manual effort, and enhance fairness.

ChatGPT^®^ (OpenAI, San Francisco, CA, USA) is used to translate natural language instructions into VBA (Visual Basic for Applications) macros, which automate the creation of on-call and daily activity schedules. The process involves collecting staff preferences via a Microsoft Excel (Microsoft Corporation, Redmond, WA, USA) sheet, followed by AI-generated VBA macros that automate the creation of the schedule, ensuring adherence to various constraints such as equitable shift distribution and prioritization of specific roles. The system was developed by a non-IT professional and does not require advanced programming skills.

The implementation of this AI-driven scheduling system resulted in a significant reduction in administrative time and increased schedule fairness, as decisions were based on clear, consistently applied rules. The system also minimized conflicts within teams, improving both organizational efficiency and staff satisfaction. However, the development of such a process was not without its challenges, particularly in terms of rule formulation and Excel cell references.

The integration of Microsoft Excel^®^ and AI LLM provides a simple and reproducible solution for hospital schedule organization, reducing administrative burden and promoting fairness. This model, which can be adapted to other sectors facing similar challenges, enables teams to retain control over the process.

## Introduction

In hospitals, especially in departments subject to continuous care obligations (night shifts, weekends, holidays), physicians’ professional lives are heavily influenced by the organization of on-call schedules and daily assignments.

Creating these schedules must account for numerous constraints, whether related to service organization (day/night distribution, continuity of care, available staff) or individual circumstances (absences, preferences, participation in conferences, etc.).

In most cases, these schedules are either prepared by the physicians themselves, thus adding a significant administrative burden at the expense of their clinical activities, or by administrative staff, who may not fully grasp all the organizational subtleties of the department, requiring multiple adjustments and revisions. In addition to the substantial administrative time this represents, creating a fair schedule remains a complex task and is often a source of frustration or tension within teams that could potentially affect the quality of life and care [1].

The aim of this article is to demonstrate that artificial intelligence-based large language models (AI LLMs), combined with widely available computer tools, can automate the creation of on-call and daily activity schedules in a fair manner, without requiring advanced programming skills or specialized software.

We describe the implementation of a semi-automated hospital scheduling system that leverages ChatGPT^®^ (OpenAI, San Francisco, CA, USA) for AI and code generation, and Microsoft Excel (Microsoft Corporation, Redmond, WA, USA) for code application and schedule management. We also evaluate its benefits and limitations. In this context, AI refers specifically to the use of the large language model (ChatGPT) to translate natural-language instructions into executable VBA (Visual Basic for Applications) code for scheduling automation.

## Technical report

Tools

Microsoft Excel^®^ was already used in our department as the main scheduling tool, and ChatGPT^®^ is a widely accessible AI interface, making the choice of tools straightforward. The objective was not to introduce new software, but simply to save time on a tedious administrative task by leveraging tools already in daily use.

Excel is a spreadsheet program that allows users to structure, process, and analyze data in a table form. It is widely adopted in professional environments, and while many are familiar with its basic functions, its advanced potential is often underutilized. Basic formulas are accessible to everyone and require no programming skills. On the other hand, macros, which automate complex sequences of actions, are written in VBA, a more technical programming language specific to Excel, often out of reach for non-IT users.

This is precisely where AI comes into play: ChatGPT can generate, correct, explain, or adapt VBA code, even from simple instructions written in natural language. This opens the door to advanced automation without requiring deep coding knowledge. One simply describes the needs (e.g., fairly distribute on-calls, respect constraints) and ChatGPT translates these requests into VBA code, ready to be copied and pasted into Excel’s macro editor. While other AI software exists, ChatGPT was chosen not only out of habit, but also for its versatility, user-friendly interface, and its ability to understand instructions in natural language, unlike other specialized software aimed mainly at expert users.

Preparation of the preference file and on-call automation

Before generating the weekly schedule, the on-call roster must be established, as it directly impacts medical availability on subsequent days. The first step involves creating a structured Excel sheet in the form of a calendar for the desired period. This file is then shared with team members so they can enter their preferences: absences for vacation or illness, conference attendance, days off, on-call requests, etc. To improve readability and prevent entry errors, each type of absence is selected via a drop-down list, paired with a color code.

Once this table is completed, it becomes the main database from which on-calls can be automatically assigned. It is crucial that ChatGPT understands the file’s structure: sheet names, data locations (columns, rows), input and output cells, explanations of preferences, and so on. This information can be described in text or supported by screenshots, making it easier for the AI to analyze.

The basic rules are then introduced progressively: strict equity in the number of on-calls per physician per quarter and year, depending on work patterns (full-time, part-time, etc.), exclusion of absentees (including those unavailable the day before a day off), priority given to on-call requests, and balanced distribution between weekdays and weekends. Once these basic rules are validated, more service-specific constraints can be added (e.g., avoid having on-call shifts for two consecutive weekends, X days off after an on-call shift, no make-up of on-call shifts in case of long-term absence, etc.).

Automation of daily assignments

Once the on-call roster is established, the daily schedule can be generated. Since the daily assignment sheet is organized differently, it is necessary to provide ChatGPT with a new, precise description of its structure (source sheet, output sheet, logic of columns and rows).

In our department, certain rules must be given priority: the daily presence of a senior physician in the emergency department and two senior physicians in the inpatient unit. Some practitioners are assigned exclusively to these hospital tasks (without outpatient activity) and should be prioritized for these roles if available. If these positions cannot be filled by permanent physicians, other practitioners are designated according to a predefined order of priority.

The AI must also take into account absences listed in the preference sheet, systematically adding a recovery day after an on-call shift, during which physicians are logically absent from daily activities. For greater clarity, all these absences and on-calls from the preference sheet are automatically transcribed into the weekly schedule. At the end of the assignment process, any physician whose cell is empty has no assignment and is therefore free to take on a consultation day (the default activity).

If it is impossible to assign according to the imposed rules (e.g., too many physicians absent, inability to fill required positions), the macro can be programmed to signal the error via a pop-up window or by coloring the relevant cell (in red, for example), allowing for manual review and adjustment if needed.

No personal medical information or confidential data is shared with the AI: the Excel files used contain only anonymized organizational data.

## Discussion

In recent years, the number of publications dedicated to AI in medicine has exploded, with most of the focus on diagnosis support, medical imaging, and clinical risk prediction [2]. However, applications of AI in hospital management and care organization remain limited. Few studies have explored the potential of automation in hospital scheduling, with one notable example being Howard et al. [3], who demonstrated that an automated tool developed in Excel could improve schedule quality and resident satisfaction. More recently, research has begun to investigate the use of ChatGPT to support hospital administrative tasks, suggesting its potential to reduce organizational burden and free up medical time for clinical practice [4].

Automating our department’s scheduling process using Microsoft Excel and ChatGPT has made decision-making more objective and significantly reduced the organizational and emotional burden. The generated schedules are now based on explicit, consistently applied rules, fostering a greater sense of fairness and limiting interpersonal conflicts. Once the initial setup was completed, subsequent updates required minimal adjustments, mainly updating dates or modifying rules, resulting in substantial time savings. At the first use, the system was associated with an approximate 50% reduction in scheduling time (from ~4 hours to ~2 hours), although this estimate remains exploratory due to limited use and ongoing macro refinement.

Comparative perspective and added value

Comparable experiences in other hospitals confirm the efficiency of scheduling automation. At C.S. Mott Children’s Hospital, automation of pediatric on-call schedules reduced planning time from 22-28 hours per month to just 4-6 hours [5]. Similarly, in Taiwan, a pediatric intensive care unit developed an Excel-based scheduling system that cut preparation time from 5 hours to 30 minutes while preserving fairness and respecting staff requests [6]. However, these studies rarely specify the time or expertise required for the initial development. In the example from Cheng et al., the system was coded by a non-IT nurse after eight days of dedicated training, highlighting both the complexity and the learning curve of traditional VBA-based systems.

Similar approaches have also been explored in emergency settings. For instance, an AI-driven forecasting system in a pediatric emergency department significantly improved shift allocation and reduced crowding [7]. However, this model primarily focused on short-term forecasting and workload redistribution within emergency units, without accounting for the limited flexibility of human resources or their integration into scheduled pediatric activities. In contrast, our approach integrates both planned and unplanned activities within a single scheduling framework, better reflecting the operational constraints of multidisciplinary hospital departments.

**Figure 1:**
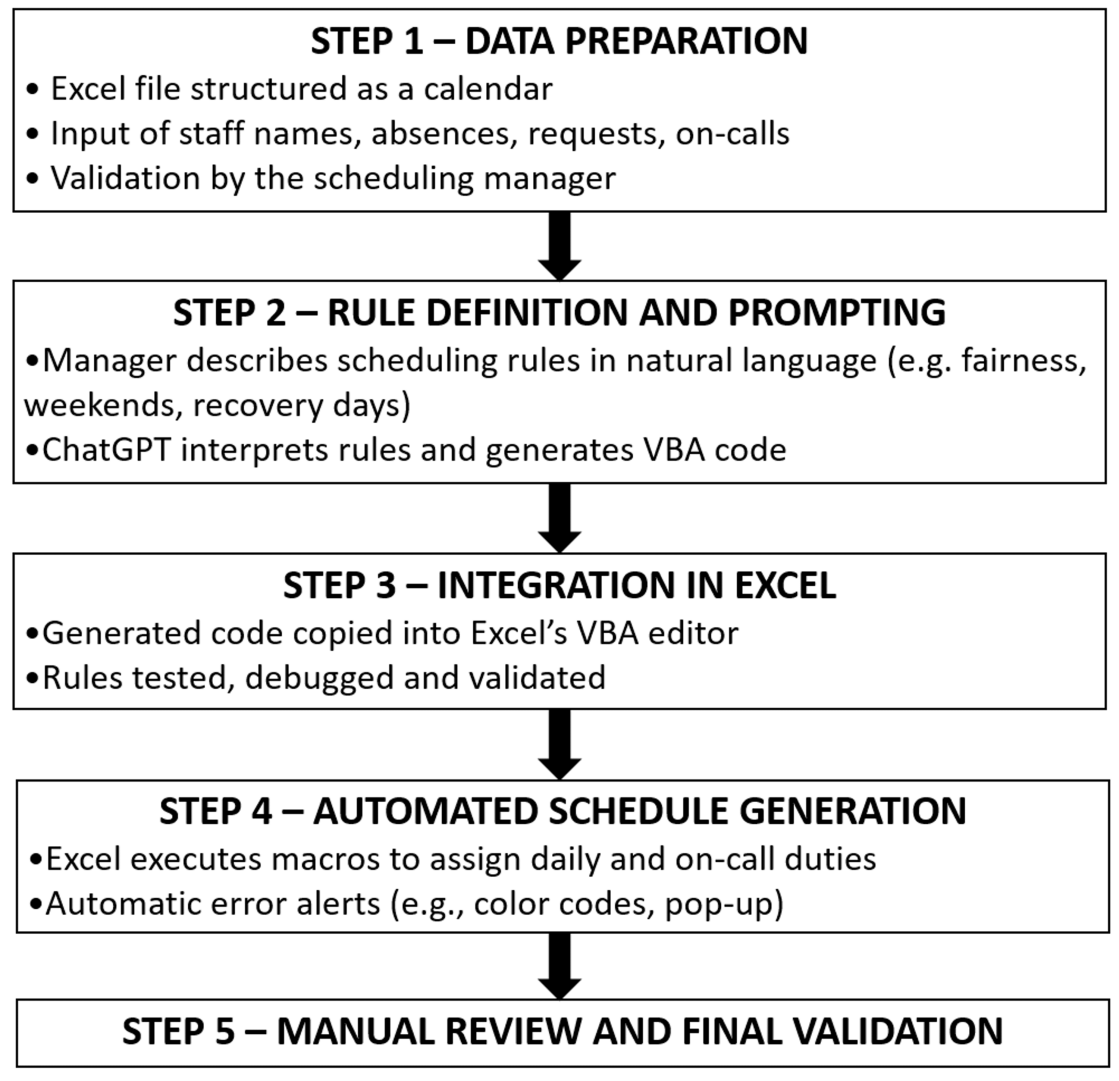
AI-Assisted Scheduling Workflow AI, artificial intelligence.

Our experience builds on and extends these findings. The originality of our approach lies in the fact that the programming was carried out directly by the person responsible for scheduling, without any programming background, using ChatGPT to generate and refine VBA macros. The entire implementation required approximately 40 hours - roughly the equivalent of one working week - and did not involve any external IT support or prior coding expertise. This autonomy represents a major step forward: it enables healthcare teams to design and maintain their own automation systems, tailored to the specificities of their service, without technical dependency. Additionally, it eliminates the need to rely on paid solutions, such as outsourcing to external management companies, resulting in a significant financial gain. Following this method (illustrated in Figure 1), the macros were successfully adapted for use in other departments, such as neonatology and the emergency departments, by the same individual responsible for the initial macro development.

Nevertheless, we do not recommend the straightforward transposition of our VBA code to other departments or institutions. In addition to the inherent challenges of translating the code into another language and the potential errors this may introduce, automation cannot substitute for human oversight. It is therefore essential to designate a dedicated resource person responsible for managing conflicts, adjusting assignments, and handling changes in constraints or staffing parameters within the macro. The process of macro development enables this individual to gain an in-depth understanding of the code, thereby facilitating more coherent and context-specific adaptations in the future. Furthermore, the system was created with specific rules tailored to the unique needs and constraints of our service, making it difficult to directly transpose without significant adjustments.

Another strength of this model lies in its accessibility and reproducibility. Unlike specialized scheduling software requiring costly training or maintenance, combining Microsoft Excel and ChatGPT allows any motivated user with basic Excel skills and sufficient time for iterative testing to progressively automate complex processes through simple natural-language interactions. ChatGPT assists step by step in generating macros, structuring tables, and debugging code, thereby lowering the technical barrier that has long limited automation in hospital management. Moreover, each iteration of ChatGPT, from versions 3.5 to 5, has markedly improved its ability to interpret scheduling rules and produce reliable VBA scripts, suggesting that such tools will become increasingly powerful and stable over time.

Beyond the hospital context, broader workforce optimization frameworks have also been developed for salaried healthcare staff [8]. While these models demonstrate strong potential for institutional management, their applicability to teams of self-employed physicians remains uncertain. Our experience suggests that AI-assisted scheduling can, however, be successfully implemented in such independent or hybrid professional structures, provided the system remains transparent, locally adaptable, and under the direct control of the scheduling manager.

Limitations

The quality of automation depends heavily on the clarity and exhaustiveness of the initial rules provided to ChatGPT. Omitting or misformulating a constraint may lead to macro execution errors or inconsistent outputs. During the initial setup, several iterations were needed before obtaining a stable and fully functional system. Errors were most often due to ambiguous rule formulation or incomplete references to Excel cell structures. These issues were progressively resolved through iterative exchanges with ChatGPT, confirming the need for human oversight during setup and validation.

In addition, ChatGPT’s performance varies significantly across versions. The free model (based on GPT-3.5) showed limitations in understanding complex logical dependencies and managing long contextual prompts. In contrast, the paid versions (GPT-4 and GPT-5) provided far better reliability, maintaining dialogue continuity and producing more robust VBA code. This variability should be taken into account by future users, as it may influence development time and the quality of results.

Finally, while automation reduces administrative workload, it does not replace human judgment. Unexpected absences, changes in staff, or new organizational priorities always require manual review and adjustment. Automation supports, but does not substitute, managerial responsibility.

## Conclusions

The combination of Microsoft Excel and AI LLM offers an accessible and reproducible way to automate hospital scheduling without programming expertise. Beyond saving time, it promotes fairness, transparency, and autonomy within medical teams. By enabling non-IT staff to build and adapt their own tools, this approach provides a promising way for improving hospital organization and, more broadly, for any field facing similar scheduling challenges.

## Figures and Tables

**Figure 2:**
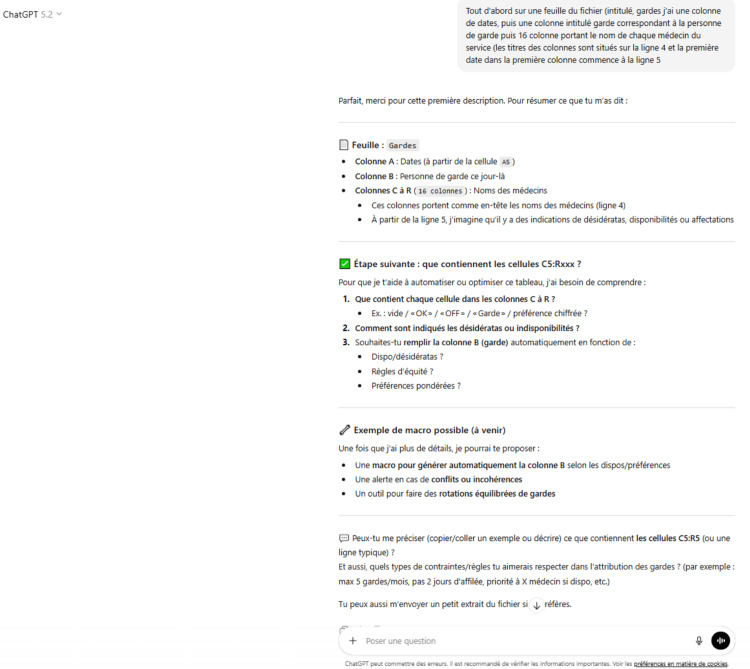
Screenshot of the beginning of the discussion with ChatGPT in the original language (French).

**Figure 3:**
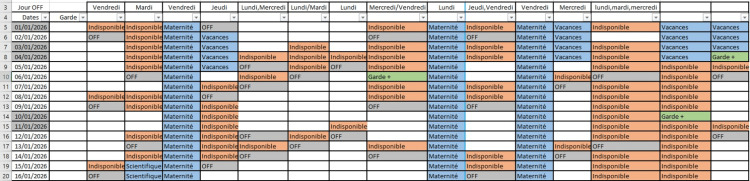
Screenshot of the Excel file structure.
